# Supplementary material for: Desaturase Activity and the Risk of Type 2 Diabetes and Coronary Artery Disease: A Mendelian Randomization Study
Source: Nutrients. 2020 Jul 28;12(8):2261. doi: 10.3390/nu12082261 (PMC7469057; doi:10.3390/nu12082261)
Supplement: Supplementary file 1 [file nutrients-12-02261-s001.zip › nutrients-12-02261-s001.pdf]

Supplementary Material

Desaturase activity and the risk of type 2 diabetes and coronary artery disease: a Mendelian randomization study

Susanne Jäger, Rafael Cuadrat, Per Hoffmann, Clemens Wittenbecher, Matthias B. Schulze*

*Department of Molecular Epidemiology, German Institute of Human Nutrition Potsdam-Rehbruecke, Nuthetal, Germany.
mschulze@dife.de (MBS)

Supplementary Tables:

Supplemental Table 1 Instruments from FADS gene cluster for D6D (R^2<0.3) and their association with T2DM or CAD

Supplemental Table 2 Instruments from FADS gene cluster for D5D (R^2<0.3) and genome-wide hits and their association with T2DM or CAD

Supplemental Table 3 Total effects of estimated desaturase activities and risk of type 2 diabetes or coronary artery disease using MR-Egger

Supplemental Table 4 Total effects of estimated delta-5-desaturase activity and risk of type 2 diabetes excluding outlying variants

Supplemental Table 5 Total effects of estimated delta-5-desaturase activity and risk of coronary artery disease excluding outlying variant

Supplemental Table 6 Association between genetic instruments used in multivariable MR and estimated desaturase activities and risk of type 2 diabetes or coronary artery disease

Supplemental Table 7 Direct effects of estimated desaturase activities and risk of type 2 diabetes or coronary artery disease using multivariable MR-Egger

Supplemental Table 8 Direct effects of estimated desaturase activities and risk of type 2 diabetes or coronary artery disease excluding outlying variants

Supplemental Table 9 Direct effects of estimated desaturase activities and risk of type 2 diabetes or coronary artery disease excluding outlying variants

Supplemental Table 10 Total and direct effects of estimated desaturase activities and risk of type 2 diabetes and coronary artery disease accounting for confounding by LD, excluding rs174602

Supplementary Figures:

Supplemental Figure 1 Flow-chart of final study population

Supplemental Figure 2 Manhattan plots visualizing GWAS results for D6D and D5D

Supplemental Figure 3 LD-Plot visualizing the degree of linkage disequilibrium between genetic instruments from *FADS* gene region evaluated within the study

Supplemental Figure 4 Scatter plots visualizing genetic associations with D6D and type 2 diabetes and coronary artery disease

Supplemental Figure 5 Scatter plots visualizing genetic associations with D5D and type 2 diabetes and coronary artery disease

Supplemental Figure 6 Radial MR plot for D5D and type 2 diabetes

Supplemental Figure 7 Scatter plots visualizing genetic associations with D5D and type 2 diabetes after outlier removal

Supplemental Figure 8 Scatter plots visualizing genetic associations with D5D and coronary artery disease after outlier removal

Supplemental Figure 9 Effect-size estimates and variant-level contribution to CAUSE test statistics for D6D and type 2 diabetes.

Supplemental Figure 10 Effect-size estimates and variant-level contribution to CAUSE test statistics for D6D and coronary artery disease.

Supplemental Figure 11 Effect-size estimates and variant-level contribution to CAUSE test statistics for D5D and type 2 diabetes.

Supplemental Figure 12 Effect-size estimates and variant-level contribution to CAUSE test statistics for D5D and coronary artery disease.

Supplemental Table 1 Instruments from *FADS* gene cluster for D6D ($R^2<0.3$) and their association with T2DM or CAD

**Instruments**				**D6D (EPIC-Potsdam)**				**T2DM (Diagram)**				**CAD (Cardiogram)**			
**rsID**	**Chr:position (Locus)**	**Effect allele/ Other allele**	**Most severe consequence**	**N**	**EAF in %**	**Beta (SE)**	**P**	**N**	**EAF in %**	**Beta (SE)**	**P**	**N**	**EAF in %**	**Beta (SE)**	**P**
rs174567*[#]	11:61593005 (*FADS2*)	G/A	Upstream gene variant	1853	33.5	-0.36 (0.03)	1.1 E-27	231420	35.0	-0.03 (0.01)	2.0e-05	335201	33.2	-0.03 (0.01)	7.09e-04
rs2851682*	11:61616012 (*FADS2*)	G/A	Intron variant	1853	10.4	-0.48 (0.05)	3.25 E-20	231420	10.0	-0.02 (0.01)	4.9e-02	335264	9.37	-0.02 (0.01)	1.42e-01
rs174602	11:61624414 (*FADS2*)	C/T	Intron variant	1853	19.6	-0.19 (0.04)	1.13 E-05	231420	20.0	-0.03 (0.01)	2.4e-04	328726	21. 9	-0.03 (0.01)	1.50e-02
rs498793	11:61624705 (*FADS2*)	C/T	Intron variant	1853	59.8	-0.17 (0.03)	6.41 E-07	231420	60.0	-0.02 (0.01)	3.3e-03	328640	59.7	-0.02 (0.01)	3.36e-02
rs7118175	11:61639358 (*FADS2*)	C/T	Downstream gene variant	1853	8.90	-0.23 (0.05)	3.97 E-05	231420	8.00	-0.01 (0.01)	3.9e-01	328601	9.70	-0.03 (0.02)	3.82e-02
rs174465[#]	11:61658800 (*FADS3*)	C/T	Synonymous variant	1853	30.8.2	-0.19 (0.04)	1.68 E-07	231420	31.0	-0.01 (0.01)	1.7e-01	335142	33.4	-0.02 (0.01)	1.07e-02

CAD, coronary artery disease; D6D, delta-6-desaturase; EAF, effect allele frequency; SE, standard error; T2DM, type 2 diabetes
[#] Not used in multivariable MR; * genome-wide significant

Supplemental Table 2 Instruments from *FADS* gene cluster for D5D (R^2<0.3) and genome-wide hits and their association with T2DM or CAD

Instruments				D5D (EPIC-Potsdam)				T2DM (Diagram)				CAD (Cardiogram)			
**rsID**	**Chr:position (Locus)**	**Effect allele/ Other allele**	**Most severe consequence**	**N**	**EAF in %**	**Beta (SE)**	**P**	**N**	**EAF in %**	**Beta (SE)**	**P**	**N**	**EAF in %**	**Beta (SE)**	**P**
rs2608073*	3:142653512 (*RP11-372E1.4*)	T/C	Intron variant	1853	8.46	-0.38 (0.06)	1.08E-09	231420	8.9	0.01 (0.01)	4.5e-01	328077	7.47	0.01 (0.02)	2.17e-01
rs174555*	11:61579760 (*FADS1*)	C/T	Intron variant	1853	28.3	-0.83 (0.03)	3.2E-140	231420	31.0	-0.02 (0.01)	4.4e-04	335215	29.1	-0.02 (0.01)	3.89e-03
rs61897792	11:61586886 (*FADS1*)	C/T	Upstream gene variant	1853	90.1	-0.35 (0.06)	1.05E-08	231420	89.0	-0.02 (0.01)	9.7e-02	321707	90.2	-0.05 (0.02)	1.60e-03
rs174582*	11:61607168 (*FADS2*)	G/A	Intron variant	1853	20.7	-0.82 (0.04)	1.95E-106	231420	22.0	-0.02 (0.01)	6.0e-03	335290	20.2	-0.03 (0.01)	1.58e-03
rs174602*	11:61624414 (*FADS2*)	C/T	Intron variant	1853	19.6	-0.35 (0.04)	1.63E-15	231420	20.0	-0.03 (0.01)	2.4e-04	328726	21.9	-0.03 (0.01)	1.50e-02
rs498793*	11:61624705 (*FADS2*)	C/T	Intron variant	1853	59.8	-0.28 (0.04)	1.47E-15	231420	60.0	-0.02 (0.01)	3.3e-03	328640	59.7	-0.02 (0.01)	3.36e-02
rs174621*	11:61630104 (*FADS2*)	A/G	Intron variant	1853	20.8	-0.60 (0.04)	1.72E-52	231420	22.0	-0.01 (0.01)	7.3e-02	323951	21.6	-0.02 (0.01)	1.41e-02
rs508768	11:61639335 (*FADS3*)	A/G	Downstream gene variant	1853	91.9	-0.28 (0.06)	3.04E-06	231420	91.0	0.02 (0.01)	9.9e-02	323995	91.3	-0.02 (0.02)	6.51e-02
rs7118175	11:61639358 (*FADS2*)	C/T	Downstream gene variant	1853	8.9	-0.32 (0.06)	4.59E-08	231420	8.00	-0.01 (0.01)	3.9e-01	328601	9.7	-0.03 (0.02)	3.82e-02
rs174462*	11:61657666 (*FADS3*)	A/G	Upstream gene variant	1853	16.2	-0.49 (0.04)	6.57E-27	231420	16.0	0.002 (0.01)	8.6e-01	335294	17.7	-0.02 (0.01)	3.95e-02
rs11644601*	16:15172118 (*RRN3*)	C/T	Intron variant	1853	26.5	-0.24 (0.04)	5.24E-10	231420	30.0	-0.02 (0.01)	2.6e-03	324904	26.8	-0.02 (0.01)	1.13e-02

CAD, coronary artery disease; D5D, delta-5-desaturase; EAF, effect allele frequency; SE, standard error; T2DM, type 2 diabetes; # Not used in multivariable MR; * genome-wide significant

Supplemental Table 3 Total effects of estimated desaturase activities and risk of type 2 diabetes or coronary artery disease using MR-Egger

		T2DM				CAD			
		**N (SNPs)***	**OR (95% CI)**	**P-value**	**Intercept (SE), p-value**	**N (SNPs)****	**OR (95% CI)**	**P-value**	**Intercept (SE), p-value**
**D6D**	instruments from *FADS*	6	1.04 (0.98 - 1.10)	0.203	0.012 (0.009), 0.185	6	1.02 (0.94 - 1.11)	0.670	0.016 (0.012) 0.172
**D5D**	instruments from *FADS*	9	1.00 (0.98 - 1.05)	0.540	0.011 (0.011), 0.306	9	1.01 (0.97 - 1.04)	0.751	0.022 (0.009), 0.018
	instruments from *FADS* and genome-wide hits	11	1.03 (1.00 - 1.06)	0.049	0.006 (0.007), 0.377	11	1.00 (0.98 - 1.03)	0.801	0.021 (0.006), 0.001

CI, confidence interval; D5D, delta-5-desaturase; D6D, delta-6-desaturase; SE, standard error

*Of the 11/7 SNPs associated with D5D/D6D, 11/7 were available in the GWAS of T2DM (Mahajan et al. 2018).
After harmonization and removal of palindromic SNPs with intermediate allele frequencies, 9/6 SNPs were included in the MR analysis on T2DM.
**Of the 11/7 SNPs associated with D5D/D6D, 10/6 were available in the GWAS of CAD (Nelson et al. 2017).
After harmonization and removal of palindromic SNPs with intermediate allele frequencies, 9/6 SNPs were included in the MR analysis on CAD.

Supplemental Table 4 Total effects of estimated delta-5-desaturase activity and risk of type 2 diabetes excluding outlying variants

	instruments from *FADS*				instruments from *FADS* and genome-wide hits			
**Method**	**N (SNPs)***	**OR (95% CI)**	**P-Value**	**Intercept (SE), p-value**	**N (SNPs)***	**OR (95% CI)**	**P-Value**	**Intercept (SE), p-value**
MR-Egger	7	1.01 (0.98 - 1.04)	0.635	0.013 (0.010), 0.219	9	1.01 (0.98 - 1.04)	0.638	0.011 (0.008), 0.163
Inverse variance weighted	7	1.03 (1.02 - 1.04)	<0.001		9	1.03 (1.01 - 1.04)	<0.001	

CI, confidence interval; SE, standard error

*two outlying instruments (rs174602, rs508768) determined by radial MR with alpha=0.05 were excluded from analyses.

Supplemental Table 5 Total effects of estimated delta-5-desaturase activity and risk of coronary artery disease excluding outlying variant

	instruments from *FADS*				instruments from *FADS* and genome-wide hits			
**Method**	**N (SNPs)***	**OR (95% CI)**	**P-Value**	**Intercept (SE), p-value**	**N (SNPs)***	**OR (95% CI)**	**P-Value**	**Intercept (SE), p-value**
MR-Egger	8	1.01 (0.96 - 1.07)	0.661	0.017 (0.015), 0.280	10	1.01 (0.98 - 1.04)	0.546	0.016 (0.008), 0.049
Inverse variance weighted	8	1.03 (1.01 - 1.05)	0.002		10	1.03 (1.01 - 1.06)	0.003	

CI, confidence interval; SE, standard error

*one possibly outlying instrument (rs61897792) determined by radial MR with alpha = 0.1 was excluded from analyses.

Supplemental Table 6 Association between genetic instruments used in multivariable MR and estimated desaturase activities and risk of type 2 diabetes or coronary artery disease

			D6D		D5D		T2DM	CAD
SNP	Chr:position (Locus)	Effect allele/ Other allele	beta (SE)	p	beta (SE)	p	OR (95% CI)	OR (95% CI)
rs2608073	3:142653512 (*RP11-372E1.4*)	T/C	-0.09 (0.06)	0.16	-0.38 (0.06)	1.08E-09	1.01 (0.99 -1.03)	1.01 (0.98 -1.05)
rs174555*	11:61579760 (*FADS1*)	C/T	-0.33 (0.03)	8.27E-22	-0.83 (0.03)	3.23E-140	0.98 (0.96 -0.99)	0.98 (0.96 -0.99)
rs61897792	11:61586886 (*FADS1*)	T/C	0.1 (0.06)	0.10	0.35 (0.06)	1.05E-08	1.02 (1.00 -1.04)	1.05 (1.02 -1.08)
rs174582*	11:61607168 (*FADS2*)	G/A	-0.18 (0.04)	2.45E-06	-0.82 (0.04)	1.95E-106	0.98 (0.96 -0.99)	0.97 (0.95 -0.99)
rs2851682*	11:61616012 (*FADS2*)	G/A	-0.48 (0.05)	3.25E-20	-0.40 (0.05)	7.50E-14	0.98 (0.96 -1.00)	0.99 (0.96 -1.01)
rs174602*	11:61624414 (*FADS2*)	C/T	-0.19 (0.04)	1.13E-05	-0.35 (0.04)	1.63E-15	0.97 (0.95 -0.99)	0.98 (0.95 -1.00)
rs498793*	11:61624705 (*FADS2*)	C/T	-0.17 (0.03)	6.416E-07	-0.28 (0.04)	1.47E-15	0.98 (0.97 -0.99)	0.98 (0.96 -1.00)
rs174621*	11:61630104 (*FADS2*)	A/G	-0.19 (0.04)	1.57E-06	-0.60 (0.04)	1.72E-52	0.99 (0.97 -1.00)	0.98 (0.96 -1.00)
rs508768	11:61639335 (*FADS3*)	G/A	0.12 (0.06)	0.04	0.28 (0.06)	3.04E-06	0.98 (0.96 -1.00)	1.02 (0.99 -1.05)
rs7118175*	11:61639358 (*FADS2*)	T/C	0.23 (0.06)	3.97E-05	0.32 (0.06)	4.59E-08	1.01 (0.99 -1.03)	1.03 (1.00 -1.06)
rs174462*	11:61657666 (*FADS3*)	A/G	-0.2 (0.04)	5.37E-06	-0.49 (0.04)	6.57E-27	1.00 (0.98 -1.02)	0.98 (0.96 -1.00)
rs11644601*	16:15172118 (*RRN3*)	C/T	0.14 (0.04)	1.75E-04	-0.24 (0.04)	5.24E-10	0.98 (0.97 -0.99)	0.98 (0.96 -1.00)

CAD, coronary artery disease; CI, confidence interval; D5D, delta-5-desaturase; D6D, delta-6-desaturase; SE, standard error; T2DM, type 2 diabetes;
* gene-wide significant for both desaturases (P < [0.05/169])

Supplemental Table 7 Direct effects of estimated desaturase activities and risk of type 2 diabetes or coronary artery disease using multivariable MR-Egger

		T2DM				CAD			
		**N (SNPs)***	**OR (95% CI)**	**P-value**	**Intercept (SE), p-value**	**N (SNPs)****	**OR (95% CI)**	**P-value**	**Intercept (SE), p-value**
**D6D**	instruments from *FADS*	10	1.02 (0.93 - 1.12)	0.632	0.009 (0.014), 0.544	10	0.98 (0.91 - 1.05)	0.503	0.021 (0.015), 0.151
	instruments from *FADS* and genome-wide hits	12	1.03 (0.95 - 1.12)	0.440	-0.005 (0.010), 0.592	12	1.02 (0.94 - 1.01)	0.679	-0.008 (0.015), 0.621
**D5D**	instruments from *FADS*	10	0.98 (0.92 - 1.05)	0.543	0.009 (0.014), 0.544	10	1.02 (0.97 - 1.07)	0.402	0.021 (0.015), 0.151
	instruments from *FADS* and genome-wide hits	12	1.01 (0.96 - 1.05)	0.827	-0.005 (0.010), 0.592	12	1.04 (0.98 - 1.01)	0.180	-0.008 (0.015), 0.621

CAD, coronary artery disease; CI, confidence interval; D5D, delta-5-desaturase; D6D, delta-6-desaturase; OR, odds ratio; SE, standard error; T2DM, type 2 diabetes

Multivariable MR Egger estimates of the effect of D5D and D6D on T2DM and CAD.
For MR Egger analysis it is crucial to align all of the SNPs in a way that they have a positive effect on the exposure. To do so we repeated the analysis and first set the genetic associations with D6D to be positive (presented in the table) and second vice versa for D5D. This adjustment made no difference to the results obtained when using only instruments from *FADS* and only minor changes were observed when using additional genome-wide hits.

Supplemental Table 8 Direct effects of estimated desaturase activities and risk of type 2 diabetes or coronary artery disease excluding outlying variants

		T2DM			CAD		
		**N (SNPs)***	**OR (95% CI)**	**P-Value**	**N (SNPs)***	**OR (95% CI)**	**P-Value**
**D6D**	instruments from *FADS*	8	1.05 (0.94 - 1.18)	0.377	8	1.02 (0.93 - 1.11)	0.741
	instruments from *FADS* and genome-wide hits	10	1.02 (0.96 - 1.08)	0.527	10	1.00 (0.94- 1.06)	0.992
**D5D**	instruments from *FADS*	8	0.98 (0.93 - 1.03)	0.439	8	1.03 (0.97 - 1.09)	0.336
	instruments from *FADS* and genome-wide hits	10	1.00 (0.97 - 1.02)	0.748	10	1.04 (1.00 - 1.08)	0.087

CAD, coronary artery disease; CI, confidence interval; D5D, delta-5-desaturase; D6D, delta-6-desaturase; OR, odds ratio; T2DM, type 2 diabetes
*two outlying instruments (rs174602, rs508768) determined by radial MR were excluded from analyses.

Supplemental Table 9 Direct effects of estimated desaturase activities and risk of type 2 diabetes or coronary artery disease excluding outlying variants

		T2DM			CAD		
		**N (SNPs)***	**OR (95% CI)**	**P-Value**	**N (SNPs)***	**OR (95% CI)**	**P-Value**
**D6D**	instruments from *FADS*	7	1.06 (0.96 - 1.17)	0.233	7	1.01 (0.94 - 1.08)	0.876
	instruments from *FADS* and genome-wide hits	9	1.02 (0.98 - 1.07)	0.313	9	1.01 (0.94 - 1.08)	0.809
**D5D**	instruments from *FADS*	7	0.98 (0.94 - 1.02)	0.277	7	1.03 (0.99 - 1.07)	0.161
	instruments from *FADS* and genome-wide hits	9	0.99 (0.97 – 1.01)	0.485	9	1.02 (0.98 - 1.05)	0.318

CAD, coronary artery disease; CI, confidence interval; D5D, delta-5-desaturase; D6D, delta-6-desaturase; OR, odds ratio; T2DM, type 2 diabetes
*three outlying instruments (rs174602, rs508768, rs61897792) determined by radial MR were excluded from analyses.

Supplemental Table 10 Total and direct effects of estimated desaturase activities and risk of type 2 diabetes and coronary artery disease accounting for confounding by LD, excluding rs174602

		T2DM			CAD		
	**Method**	**N (SNPs)**	**OR (95% CI)**	**P-Value**	**N (SNPs)**	**OR (95% CI)**	**P-Value**
**D6D**	IVW*	2 †	1.10 (1.03 - 1.17)	0.006	2 †	1.11 (1.02 - 1.22)	0.002
	MVIVW*	2 †	0.71 (0.18 - 2.73)	0.617	2 †	1.32 (0.02 - 8.82)	0.778
**D5D**	MVIVW*	2 †	1.33 (0.51 - 3.50)	0.562	2 †	0.90 (0.26 - 3.12)	0.866

* fixed effect model

† including only *FADS1*-independent (R^2<0.31) *FADS2* variants (rs498793, rs7118175)

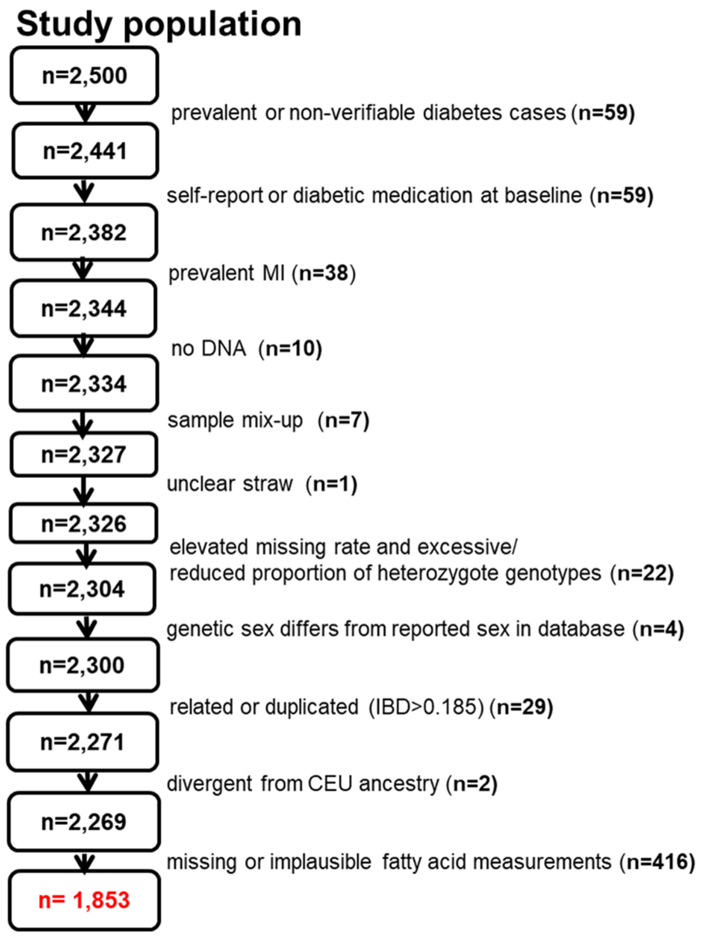

Supplemental Figure 1 Flow-chart of final study population

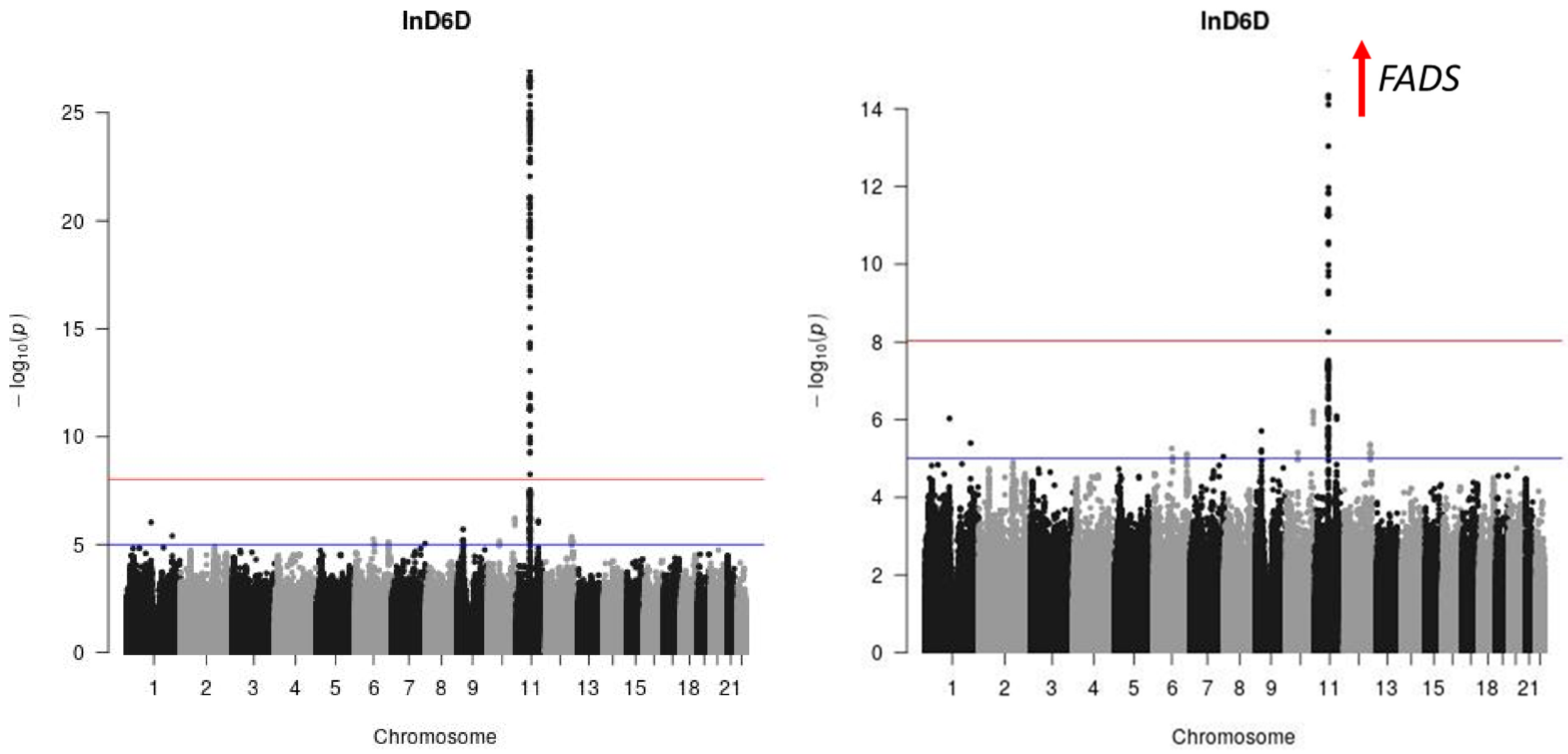
lnD6D
lnD6D
FADS
$-\log_{10}(p)$
Chromosome
25
20
15
10
5
0
14
12
10
8
6
4
2
0
1
2
3
4
5
6
7
8
9
11
13
15
18
21

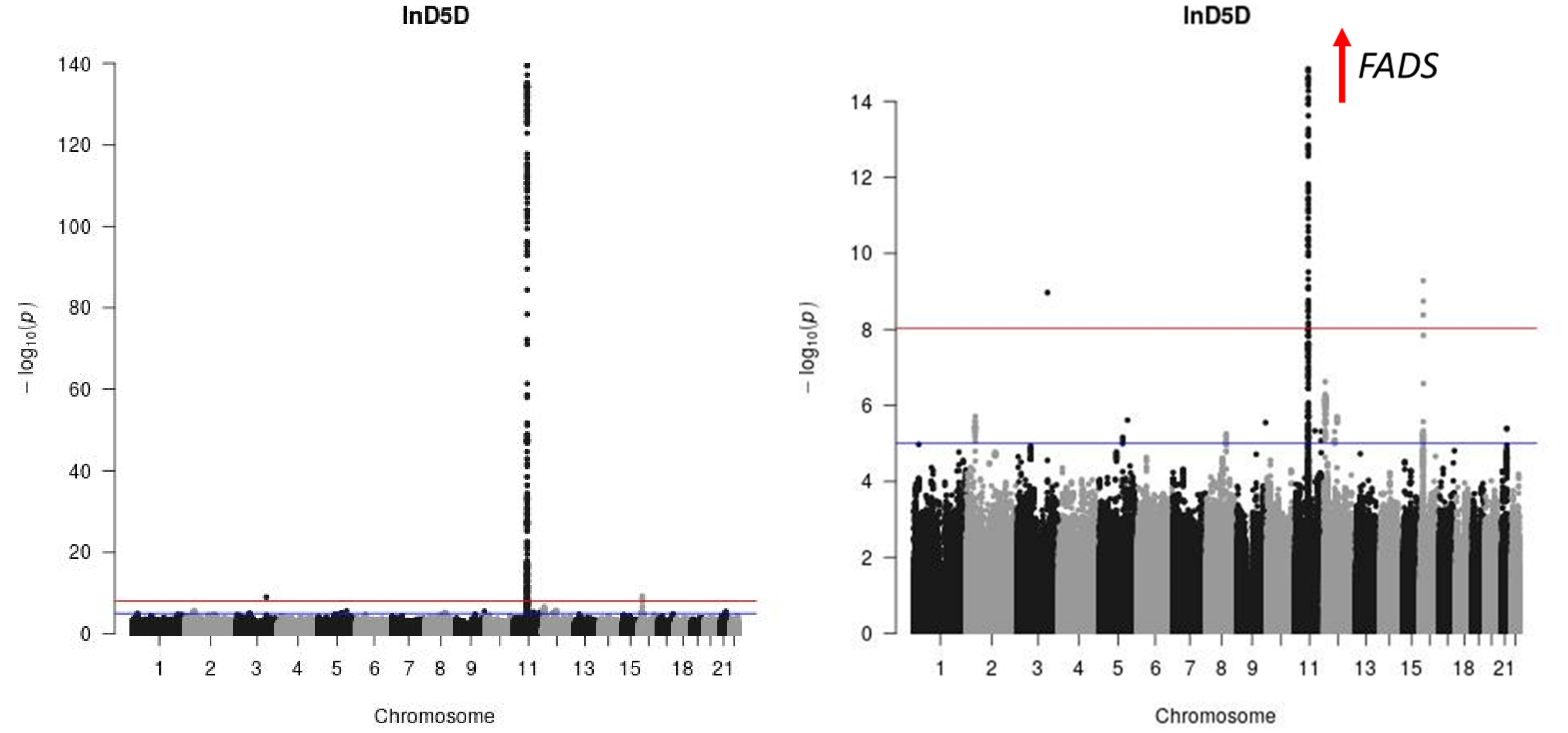

Supplemental Figure 2 Manhattan plots visualizing GWAS results for D6D and D5D

The Manhattan plots depict the distribution of association test statistics versus genomic position, with chromosomes 1 to 22 on the X axis. The Y axis represents log10-scaled p-values. The horizontal red line indicates the genome-wide significance threshold (9.36×10^{-9}); the horizontal blue line depicts the suggestive significance threshold (1×10^{-5}). Plots on the left visualize all GWAS results while for plots on the right the y-axis was truncated at $-\log_{10}(p)=15$ to standardize the y-axis for D6D and D5D.

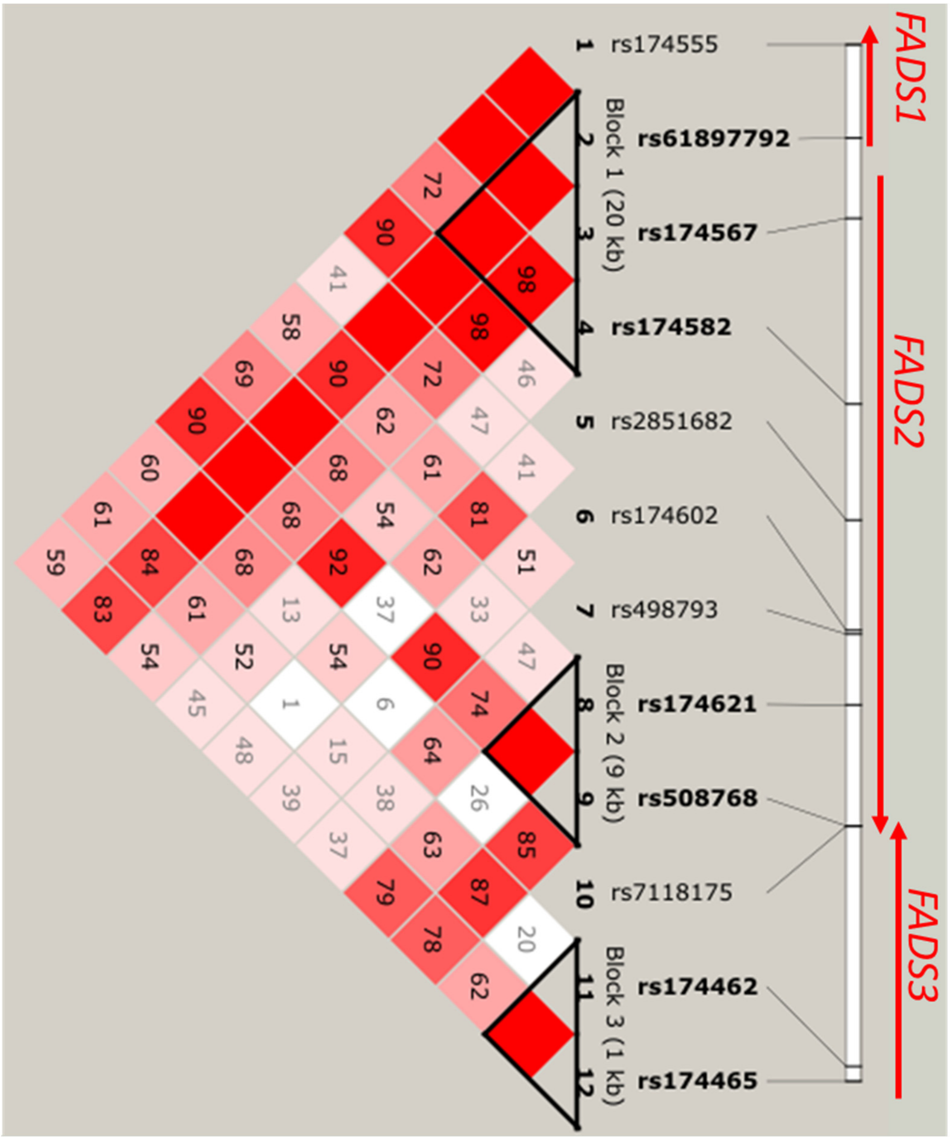

Supplemental Figure 3 LD-Plot visualizing the degree of linkage disequilibrium between genetic instruments from *FADS* gene region evaluated within the study

Haploview analysis for D' and r^2 pairwise measures of LD between genetic instruments from the EPIC-Potsdam study population. Blocks were defined using default GAB method.
D' values and confidence levels (LOD) are represented as bright red for D' = 1, LOD≥2; shades of pink/red for high D'<1, LOD≥2; white for D'<1, LOD<2.
The numbers within the squares represent the D' scores for pairwise LD.

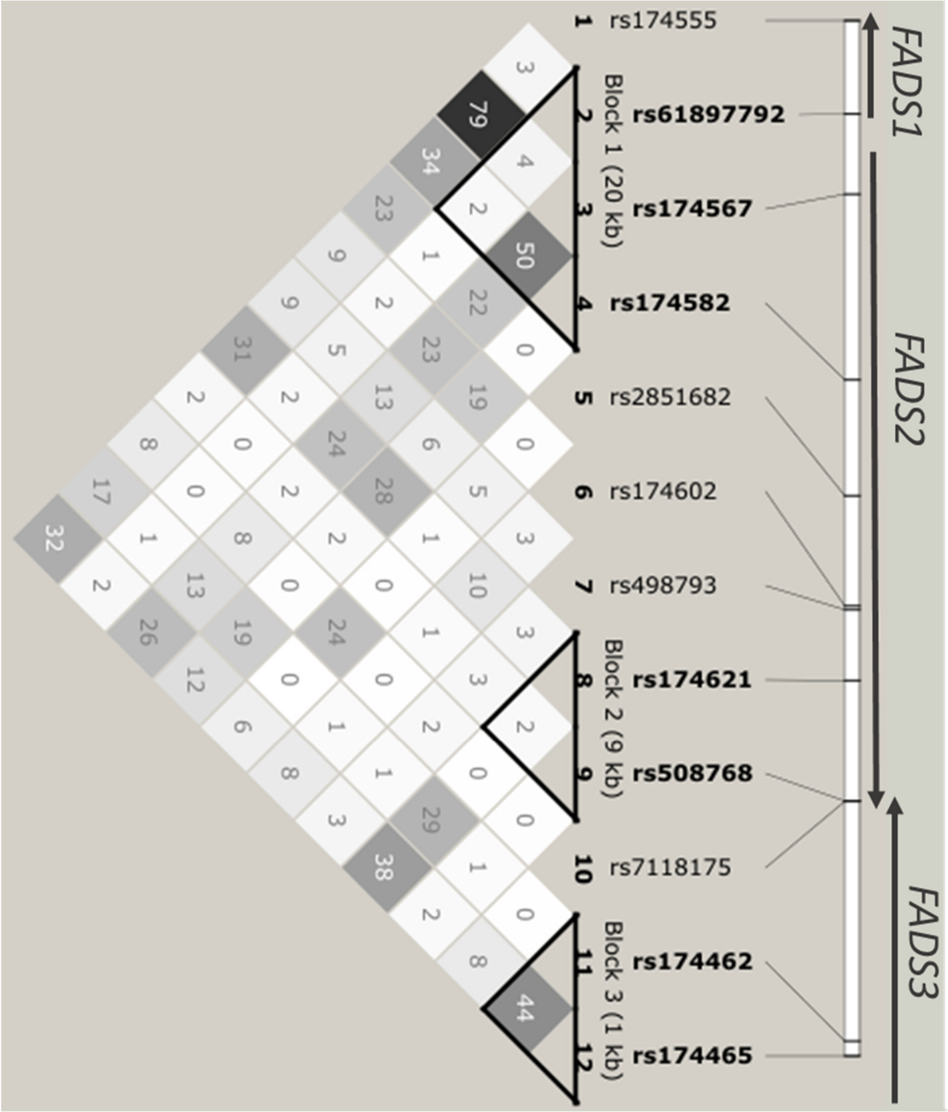

r^2 values are represented as black for $r^2 = 1$, white for $r^2 = 0$, with intermediate values for $0 < r^2 < 1$ indicated by shades of grey. The numbers within the squares represent the r^2 scores for pairwise LD.

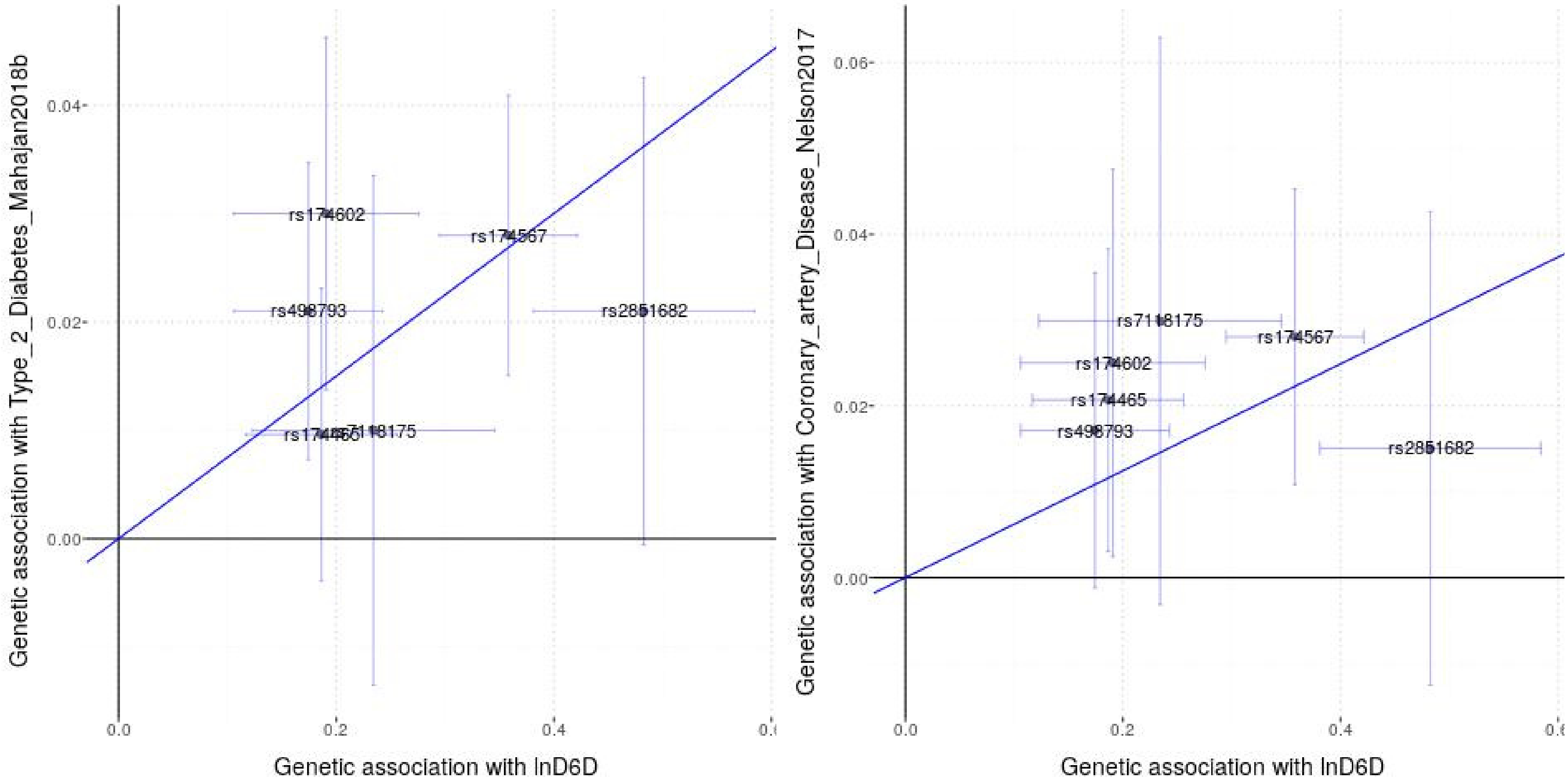

Supplemental Figure 4 Scatter plots visualizing genetic associations with D6D and type 2 diabetes (left panel) and coronary artery disease (right panel)

Each point represents the per allele associations of a single genetic variant within *FADS* (lines from each point are 95% confidence intervals for the associations). The slope of the regression represents the estimate of the causal effect of the exposure on the outcome determined by IVW method.

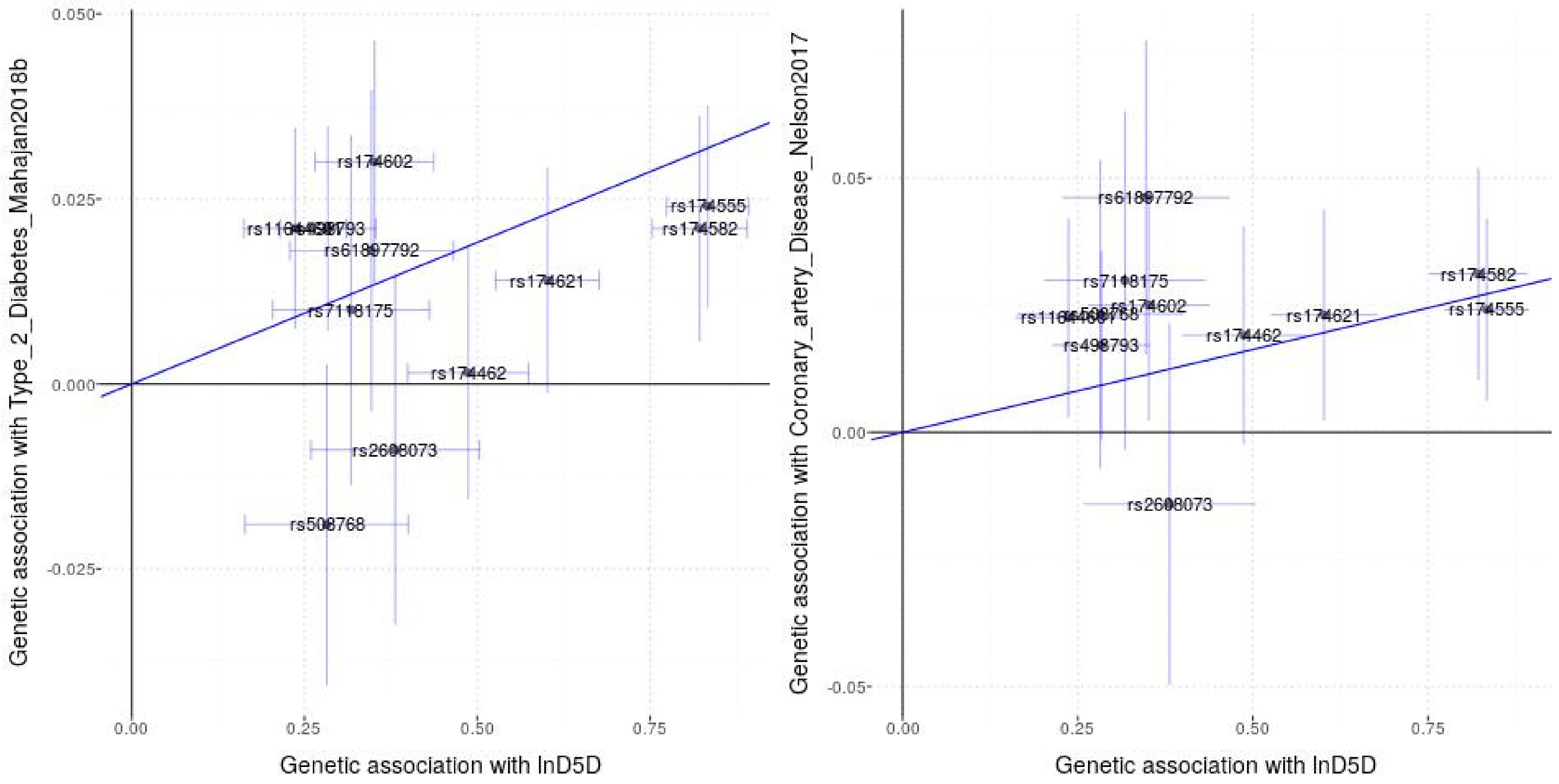

Supplemental Figure 5 Scatter plots visualizing genetic associations with D5D and type 2 diabetes (left panel) and coronary artery disease (right panel)

Each point represents the per allele associations of a single genetic variant within *FADS* and genome-wide significant hits (lines from each point are 95% confidence intervals for the associations). The slope of the regression represents the estimate of the causal effect of the exposure on the outcome determined by IVW method.

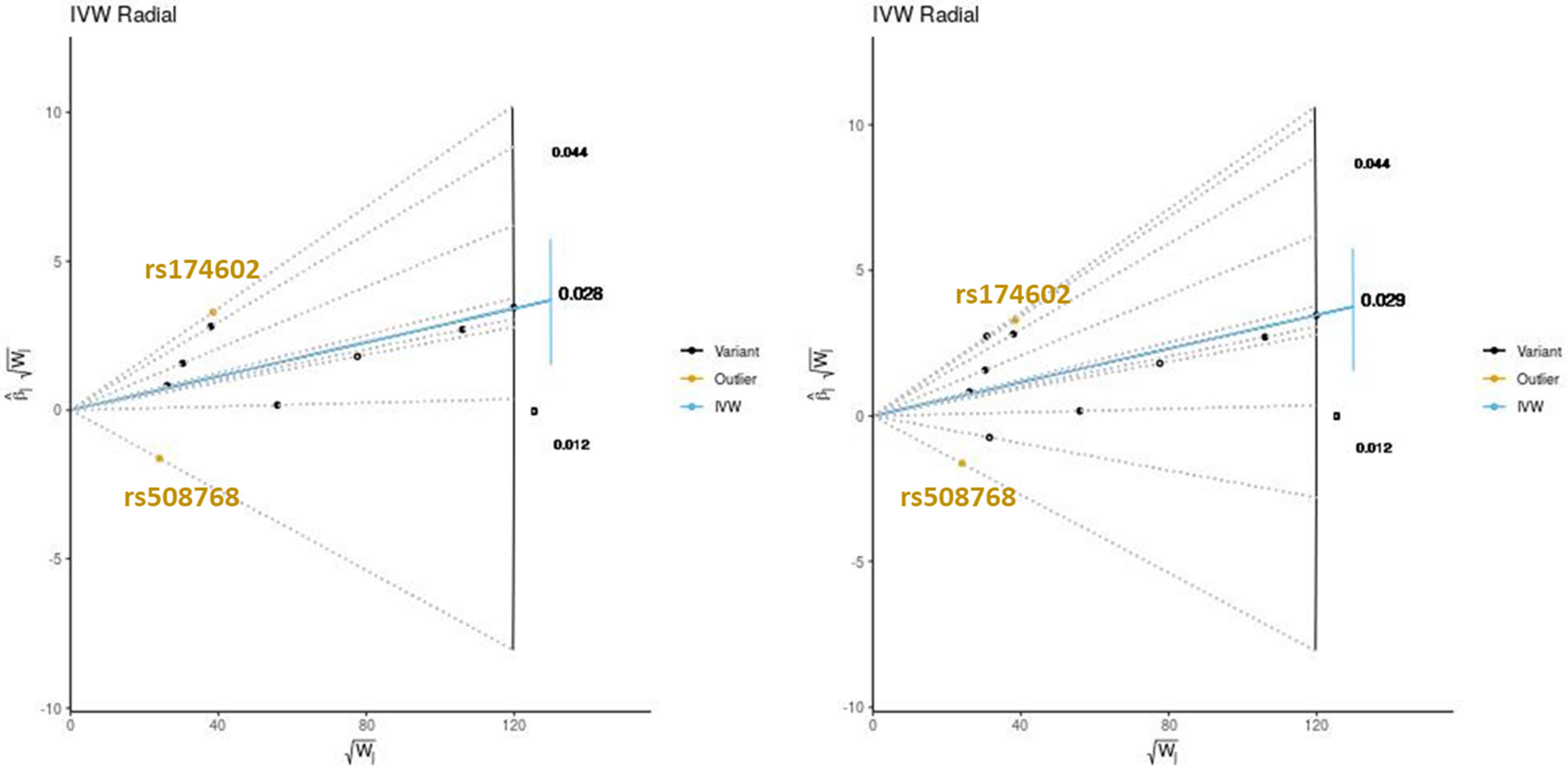

Supplemental Figure 6 Radial MR plot for D5D and type 2 diabetes

Radial curve displays the ratio estimate for each genetic variant (in black), as well as the overall inverse variance weighted (IVW) estimate (in blue). Data points with large contributions to Cochran's Q statistic are shown in yellow, with an alpha of 0.05. The graph in the left panel visualizes all included *FADS* instruments while the graph in the right panel includes additional genome-wide hits.

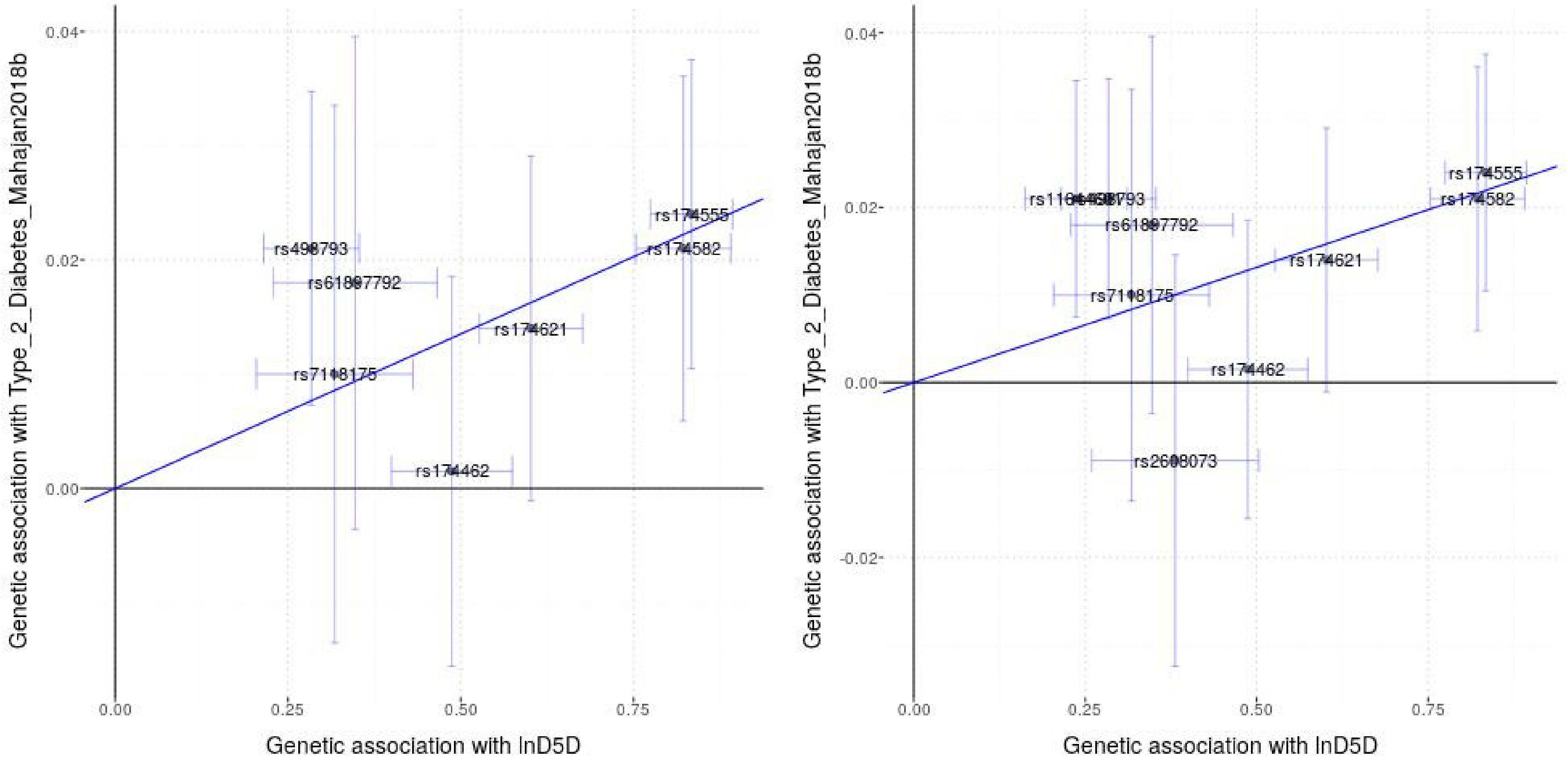

Supplemental Figure 7 Scatter plots visualizing genetic associations with D5D and type 2 diabetes after outlier removal

Each point represents the per allele associations of a single genetic variant (lines from each point are 95% confidence intervals for the associations). The slope of the regression represents the estimate of the causal effect of the exposure on the outcome determined by IVW method. The graph in the left panel visualizes all included *FADS* instruments while the graph in the right panel includes additional genome-wide hits.

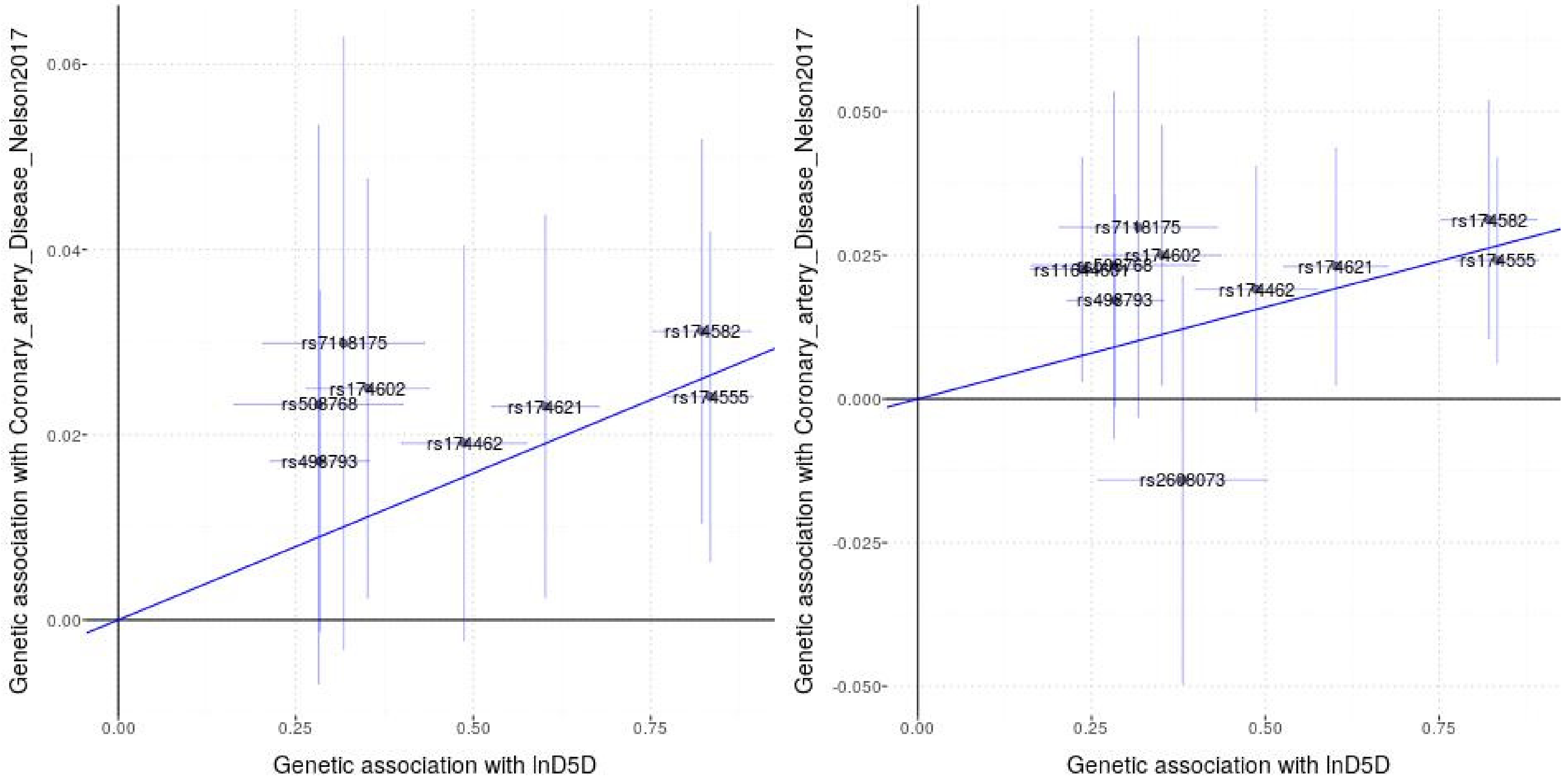

Supplemental Figure 8 Scatter plots visualizing genetic associations with D5D and coronary artery disease after outlier removal

Each point represents the per allele associations of a single genetic variant (lines from each point are 95% confidence intervals for the associations). The slope of the regression represents the estimate of the causal effect of the exposure on the outcome determined by IVW method. The graph in the left panel visualizes all included *FADS* instruments while the graph in the right panel includes additional genome-wide hits.

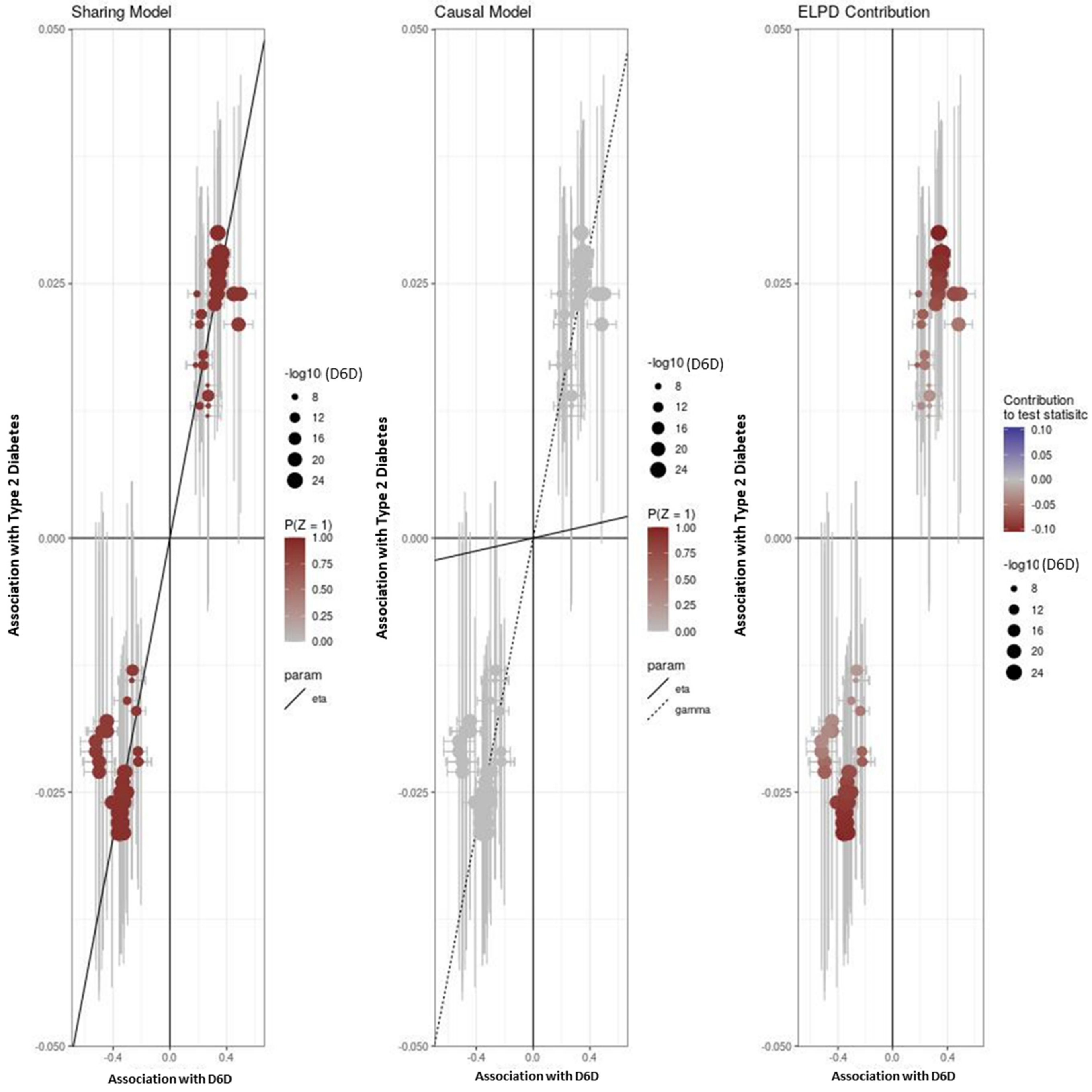

	model1	model2	delta_elpd	se_delta_elpd	z	p
1	null	sharing	-69	6.70	-10.0	4.2e-25
2	null	causal	-78	7.60	-10.0	1.2e-24
3	sharing	causal	-9	0.96	-9.3	5.7e-21

model	gamma	eta	q
Sharing	NA	0.07 (0.06, 0.09)	0.84 (0.72, 0.92)
Causal	0.07 (0.06, 0.08)	0 (-0.2, 0.25)	0.02 (0, 0.23)

Supplemental Figure 9 Effect-size estimates and variant-level contribution to CAUSE test statistics for D6D and type 2 diabetes.

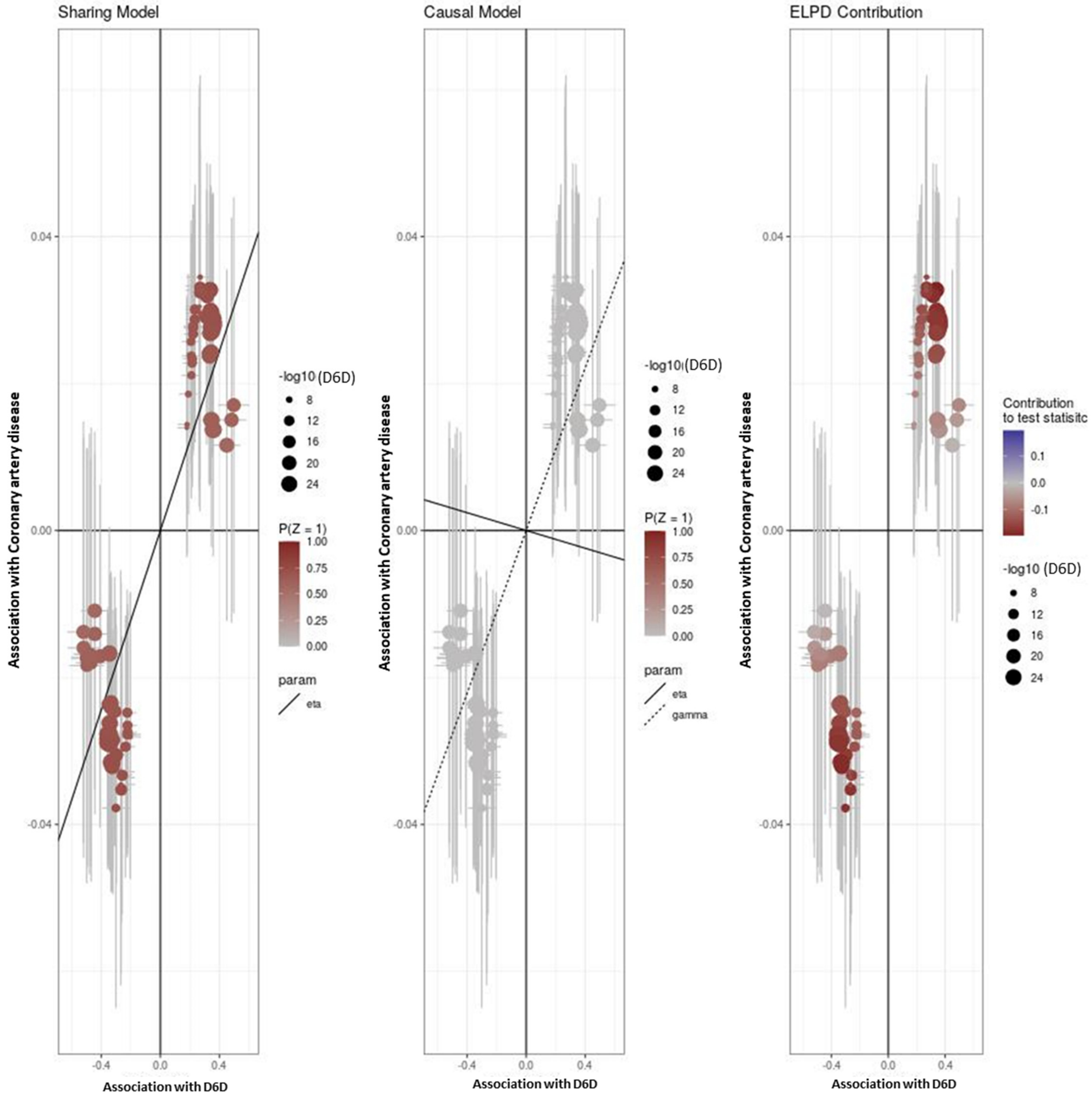

	model1	model2	delta_elpd	se_delta_elpd	z	p
1	null	sharing	-19.0	3.3	-5.6	1.3e-08
2	null	causal	-27.0	4.9	-5.4	2.9e-08
3	sharing	causal	-8.2	1.6	-5.1	1.9e-07

model	gamma	eta	q
Sharing	NA	0.06 (0.04, 0.08)	0.59 (0.35, 0.78)
Causal	0.06 (0.04, 0.07)	-0.01 (-0.23, 0.14)	0.03 (0, 0.24)

Supplemental Figure 10 Effect-size estimates and variant-level contribution to CAUSE test statistics for D6D and coronary artery disease.

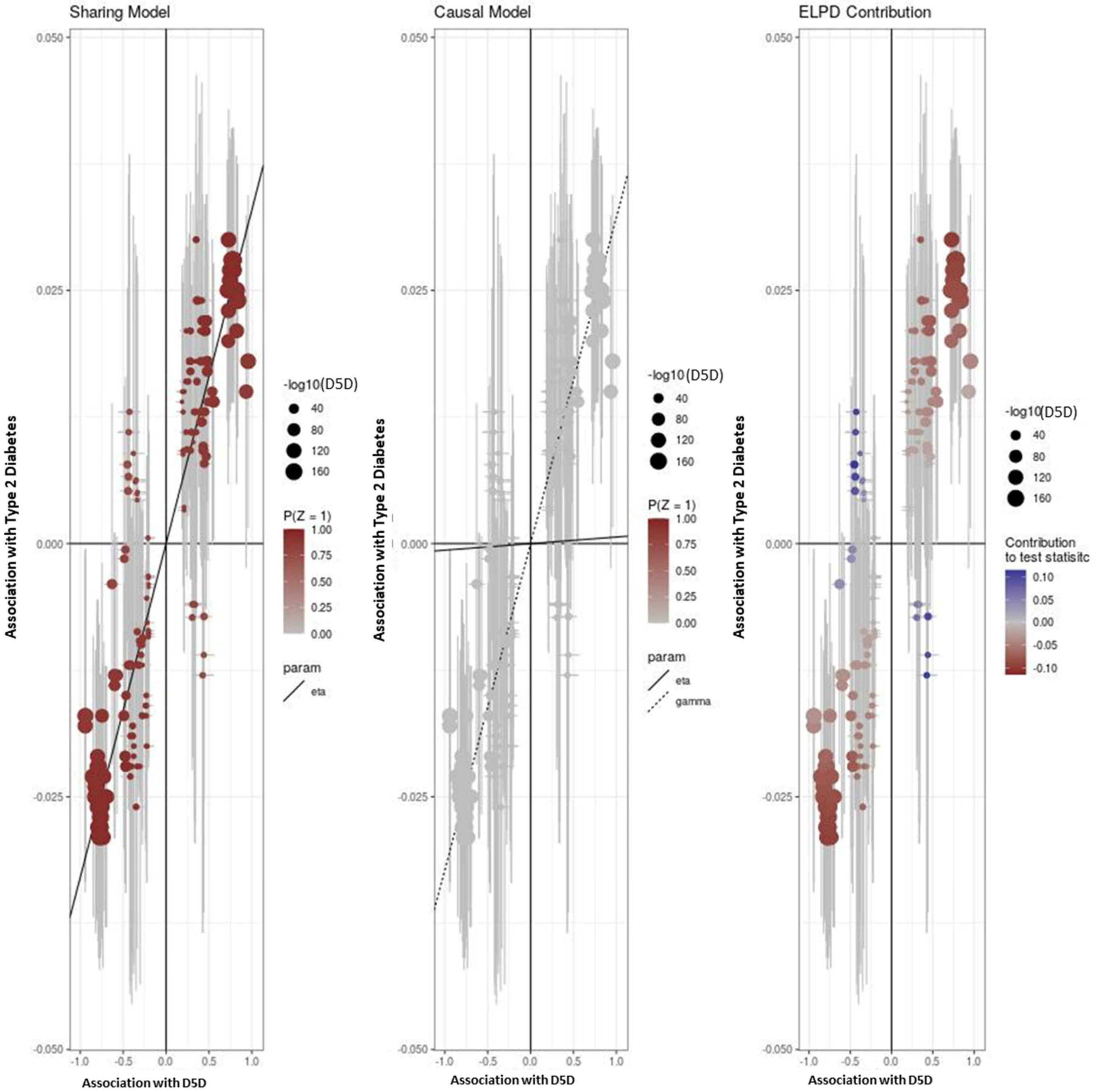

	model1	model2	delta_elpd	se_delta_elpd	z	p
1	null	sharing	-78.0	7.40	-11.0	1.5e-26
2	null	causal	-87.0	8.30	-11.0	4.0e-26
3	sharing	causal	-8.9	0.94	-9.5	7.3e-22

model	gamma	eta	q
Sharing	NA	0.03 (0.03, 0.04)	0.85 (0.75, 0.92)
Causal	0.03 (0.03, 0.04)	0 (-0.15, 0.17)	0.02 (0, 0.23)

Supplemental Figure 11 Effect-size estimates and variant-level contribution to CAUSE test statistics for D5D and type 2 diabetes.

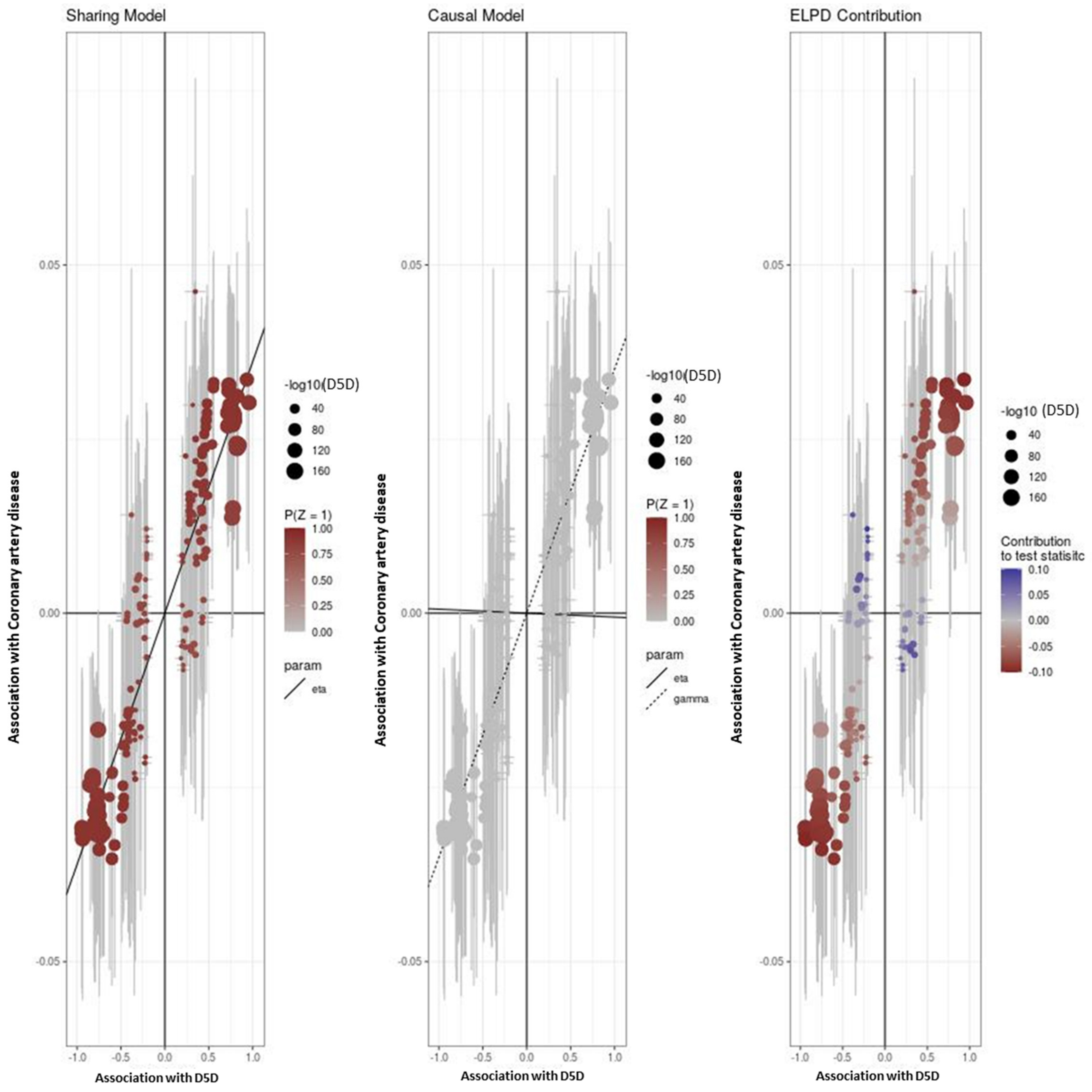

	model1	model2	delta_elpd	se_delta_elpd	z	p
1	null	sharing	-62.0	6.4	-9.7	2.1e-22
2	null	causal	-71.0	7.5	-9.5	8.3e-22
3	sharing	causal	-8.7	1.0	-8.5	9.6e-18

model	gamma	eta	q
Sharing	NA	0.04 (0.03, 0.04)	0.82 (0.7, 0.91)
Causal	0.04 (0.03, 0.04)	0 (-0.18, 0.18)	0.02 (0, 0.23)

Supplemental Figure 12 Effect-size estimates and variant-level contribution to CAUSE test statistics for D5D and coronary artery disease.
