# Supplementary figures and images for: Desaturase Activity and the Risk of Type 2 Diabetes and Coronary Artery Disease: A Mendelian Randomization Study
Source: Nutrients. 2020 Jul 28;12(8):2261. doi: 10.3390/nu12082261 (PMC7469057; doi:10.3390/nu12082261)

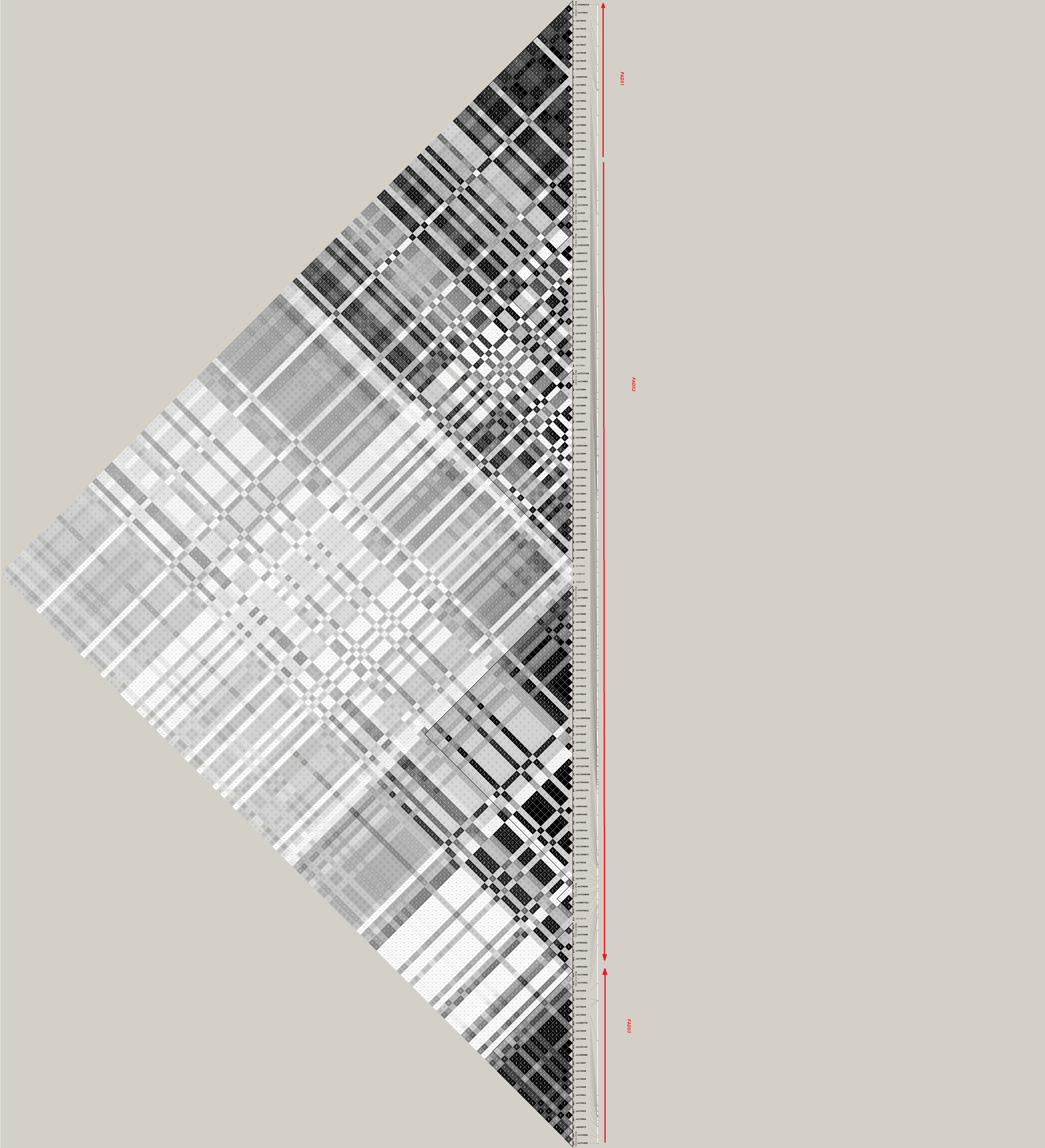

Supplement: Supplementary file 1 [file nutrients-12-02261-s001.zip › nutrients-847762-figure 13.png]
